# Benefits from early trial involvement in metastatic colorectal cancer: outcomes from the phase I unit at the Sarah Cannon Research Institute UK

**DOI:** 10.1016/j.esmogo.2024.100054

**Published:** 2024-04-17

**Authors:** R. Woodford, S. Luo, E. Ignatova, A. Cammarota, J. Choy, R. Grochot, A. Williams, T. Arkenau, E. Fontana

**Affiliations:** 1 Sarah Cannon Research Institute, London, UK; 2 National Health and Medical Research Council Clinical Trials Unit, University of Sydney, Camperdown, Australia; 3 University College London, London, UK; 4 Department of Biomedical Sciences, Humanitas University, Milan, Italy

**Keywords:** colorectal cancer, clinical trials, precision oncology, drug development unit, phase I

## Abstract

**Background:**

Metastatic colorectal cancer (CRC) is associated with poor overall survival (OS) and limited activity of approved therapeutics following two standard lines of chemotherapy. Participation in phase I trials could offer an alternative treatment option; however, benefit from participation remains unclear.

**Materials and methods:**

Medical records of patients enrolled in phase I trials at the Sarah Cannon Research Institute UK between October 2011 and July 2022 were reviewed. Patients who had received at least one dose of investigational therapy were included. Patient demographics, tumor histopathologic and molecular characteristics, clinical outcomes, including objective response rate (ORR) and clinical benefit rate (CBR), and drug details were assessed using descriptive statistics and univariable and multivariable analyses.

**Results:**

Of 1796 patients screened for phase I trials, 80 CRC patients from 31 phase I trials of 27 distinct investigational agents were included in the analysis. Overall, 53.8% were men, median age was 59 years (range 31-80 years) and median number of prior lines was 2 (range 1-6 prior lines). Median follow-up was 7 months (range 0.3-70.8 months). ORR was 7% [95% confidence interval (CI) 3.3% to 15.7%] and CBR 47% (95% CI 40.3% to 62%) across all trials. Median OS was 16.8 months (95% CI 8.8-22.0 months). The 12-month survival rate was 58%. Subgroup assessment demonstrated better outcomes for subjects receiving immunotherapies, while multivariable logistical regression demonstrated increased OS for surgery on the primary tumor [hazard ratio (HR) 0.05 (95% CI 0.00-0.69), *P* = 0.03], low lymphocyte/monocyte ratio [HR 0.45 (95% CI 0.20-0.95), *P* = 0.04] and left-sidedness [HR 0.10 (95% CI 0.14-0.70), *P* = 0.02].

**Conclusions:**

Phase I trials may provide relevant benefits for patients with refractory CRC with comparable survival to third-line therapies. Early consideration of phase I involvement may provide expedited access to potential future standard-of-care options.

## Highlights

• Phase I trials are a valuable option for chemorefractory CRC.
• Outcomes are comparable with approved third-line treatments (ORR 7%, CBR 47%).
• Left-sidedness, adjuvant therapy and primary surgery noted improved OS.

## Introduction

Colorectal cancer (CRC) is the most common gastrointestinal tract cancer and the second highest cause of cancer-related deaths in Europe in 2018.^1^ For those diagnosed with metastatic disease, 5-year relative survival following diagnosis remains generally poor, being 13% and 17% for colon and rectal cancer, respectively.^2^ These rates have improved with the ability to target oncogenic pathways with epidermal growth factor receptor,^3^ vascular endothelial growth factor^4^ and BRAF inhibition,^5^ as well as with the use of anti-programmed cell death protein 1 (PD-1) therapy for mismatch repair deficient (MMRd) tumors.^6^

In later lines, molecularly targeted approaches against additional alterations remain only applicable to a restricted group and despite these alternative options, most patients will progress and require further lines of treatment. Phase I trials, typically small, uncontrolled studies aimed at establishing the recommended dose and schedule of investigational therapies for subsequent phase II trials, may offer a late-line option for patients fit enough to consider further treatment after all conventional lines have been exhausted.^7^, ^8^, ^9^, ^10^ Eligibility criteria for these studies increasingly include selection for molecular alterations that may enrich for response.^11^ As a consequence of this selection, patients gain access to molecular testing in settings in which this may not otherwise be available, while also obtaining early access to drugs that have the potential to become a future standard of care.

Nevertheless, the path for therapeutics from phase I assessment to regulatory approval can be problematic, and matching of patients to trials remains an imprecise art wherein benefit is often unclear. By leveraging the large trial portfolio of the Sarah Cannon Research Institute UK (SCRI UK), a leading clinical research network specializing in oncology clinical trials and early drug development, we undertook a retrospective review of all patients with metastatic CRC participating in phase I clinical trials at this site to assess patient outcomes from trial participation.

## Materials and methods

We reviewed electronic medical records of patients with metastatic CRC enrolled on to phase I trials at SCRI UK between October 2011 and July 2022. Patients were eligible for assessment if they received at least one dose of trial drug following trial enrollment. Those who were not eligible based on tumor characteristics or performance status or were withdrawn for any reason before investigational therapeutic administration were excluded from assessment.

Pertinent patient and tumor characteristics, laboratory values, molecular profile, lines of prior treatment and trial therapy were independently collected by two investigators (SL, EI).

Patient-level outcomes were extracted to estimate objective response rate (ORR), clinical benefit rate (CBR), progression-free survival (PFS) and overall survival (OS). OS was defined as the time from start of first trial treatment to death. PFS was defined as the time from start of treatment to progression or death, whichever came first. ORR and CBR were assessed by site investigators, according to RECIST version 1.1, as the percentages of patients achieving a response and those responding or with stable disease, respectively.

### Subgroup analysis

Trial therapy was categorized into five groups according to the targeted pathway: rat sarcoma viral oncogene homolog (RAS)/v-raf murine sarcoma viral oncogene homolog B1 (RAF) pathway inhibitors (including BRAF, MEK, PI3K, MEK/FAK and RAS inhibitors), ataxia-telangiectasia and Rad3-related protein (ATR) inhibitors, fibroblast growth factor receptor (FGFR) inhibitors, immunotherapies (including anti-PD-1 monoclonal antibodies and immune-cell activation agents) and others (including mesenchymal epithelial transition inhibitors, fatty acid synthase inhibitors, romidepsin histone deacetylase inhibitors, anaplastic lymphoma kinase, Csk-homologous kinase and CBL-B inhibitors as well as cytotoxic agents, groups too small to consider individually). For each of these groups, outcomes were assessed separately for ORR, CBR, PFS and OS.

### Statistical analysis

Descriptive statistics were used to summarize patient characteristics. Patients enrolled on to multiple phase I trials were considered independently for each subsequent trial and were able to be counted multiple times in the survival outcome analysis. These patients were only considered once in the assessment of patient demographics.

The log-rank and Fisher’s exact tests were used to assess the association between variables. Cut-points for univariable and multivariable analyses of characteristics were designated according to published prognostic modeling.^12^ The Kaplan–Meier method was used to estimate time-to-event endpoints, with Cox regression used to estimate the hazard ratio (HR) and to backward-select for prognostic factors for PFS and OS where a *P* value of <0.05 was considered statistically significant.^13^ A waterfall plot analysis was used to demonstrate the best radiological response for each patient.

All analyses were undertaken using IBM SPSS Statistics v27.0 and StataSE 15.2 (StataCorp LLC, College Station, TX).

## Results

**Figure 1:**
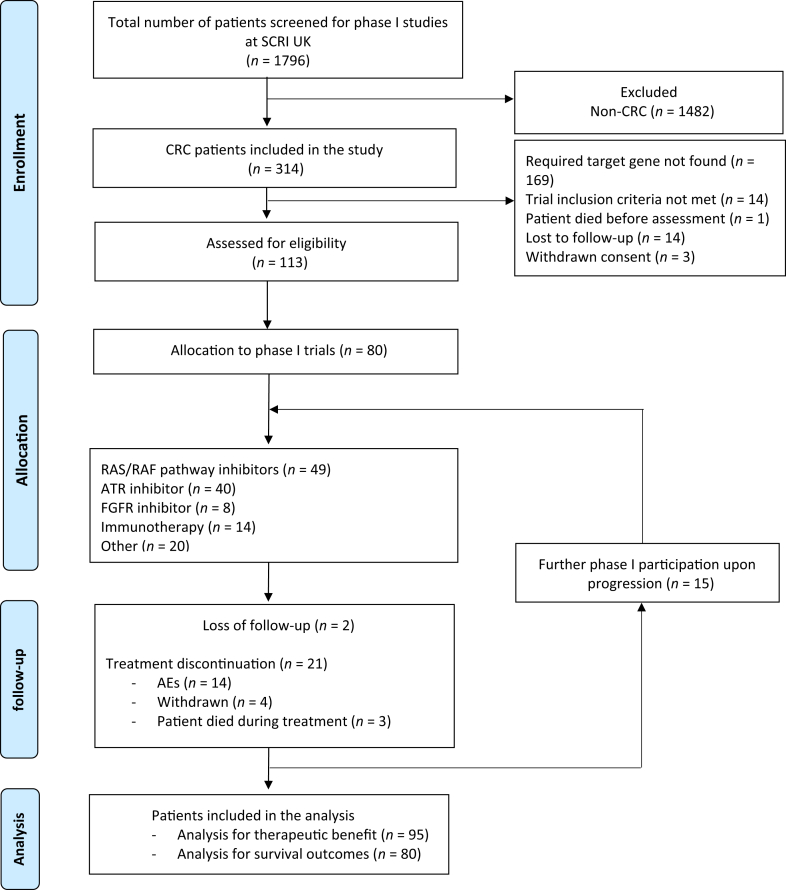
**CONSORT diagram for patient selection.***n* represents the total number of trial cases included in the analysis. Patients who participated in multiple trials were designated as separate cases. AEs, adverse events; ATR, ataxia-telangiectasia and Rad3-related protein; CONSORT, Consolidated Standards of Reporting Trials; CRC, colorectal cancer; FGFR, fibroblast growth factor receptor; RAF, v-raf murine sarcoma viral oncogene homolog B1; RAS, rat sarcoma viral oncogene homolog; SAE, severe adverse event; SCRI, Sarah Cannon Research Institute.

Of the 1796 patients enrolled into phase I trials at SCRI UK, 315 had a diagnosis of CRC. A total of 80 patients enrolled across 31 phase I clinical trials between October 2011 and July 2022 met the eligibility criteria for assessment. Baseline patient and tumor characteristics are summarized in Table 1. Eleven patients were enrolled in more than one trial, making a total of 95 cases assessable for response and PFS and 80 patients assessable for OS. The maximum number of trials that a single patient was enrolled in was four. The process of screening and patient selection is outlined in Figure 1.

**Table 1:** Baseline patient and tumor characteristics

Characteristics	*n* (%)
Age, years	
Median [range]	59 [31-80]
Sex	
Male	43 (53.8)
Female	37 (46.3)
ECOG	
0	50 (61.7)
1	30 (37.5)
Number of metastatic sites before trial entry	
Median [range]	2 [1-6]
≤2	52 (65.0)
>2	28 (35.0)
Metastatic sites	
Liver	50 (62.5)
Lung	39 (48.8)
Lymph node	31 (38.8)
Peritoneum	28 (35.0)
Bone	8 (10.0)
Brain	2 (2.5)
Others	15 (18.8)
Surgery	
Primary tumor	56 (70.0)
Metastasectomy	20 (25.0)
Previous systemic treatment	
Adjuvant	25 (31.2)
Neoadjuvant	9 (11.3)
Palliative alone	45 (56.3)
Number of prior therapies	
Median [range]	2 [0-5]
0	2 (2.5)
1-2	54 (67.5)
≥3	24 (30.0)
Duration of oxaliplatin	
>6 months	15 (18.5)
≤6 months	66 (81.5)
Tumor location	
Colon	57 (71.3)
Rectum	21 (26.3)
Tumor sidedness	
Left colon	29 (52.7)
Right colon	26 (47.3)
Stage at diagnosis	
II	7 (8.8)
III	30 (37.5)
IV	24 (30.0)
NA	19 (23.5)
Histology	
Adenocarcinoma	65 (80.2)
Mucinous adenocarcinoma	11 (13.8)
Signet ring cell carcinoma	1 (1.3)
Desmoplastic adenocarcinoma	1 (1.3)
Goblet cell carcinoma	1 (1.3)
Molecular profile	
RAS mutation	37 (46.3)
BRAF mutation	17 (21.3)
RAS/RAF wild type	14 (17.5)
NA	12 (15.0)
MSI status	
Stable	26 (32.5)
High	10 (12.5)
NA	44 (55.0)

ECOG, Eastern Cooperative Oncology Group; MSI, microsatellite instability; RAF, v-raf murine sarcoma viral oncogene homolog B1; RAS, rat sarcoma viral oncogene homolog; NA, not available.

Next-generation sequencing with an extended panel was available for 30 patients (37.5%). Mutations of interest and panel details are designated in Supplementary Table S1, available at https://doi.org/10.1016/j.esmogo.2024.100054.

The 31 clinical trials involved used 27 distinct agents, categorized by pathway and mechanism of action. Forty-nine patients (51.6%) received RAS/RAF pathway inhibitors, 6 (6.3%) received ATR inhibitors, 6 (6.3%) received FGFR inhibitors, 14 (14.6%) received immunotherapies and 20 (20.8%) received other therapies. Of the total population, 37 patients received therapies for corresponding genomic alterations, designated the ‘biomarker-selected group’. This included 21 patients with RAS/RAF alterations receiving RAS/RAF inhibitors, 7 patients with microsatellite instability (MSI)-high/MMRd tumors receiving immunotherapies, 2 patients with FGFR fusions, 5 with mutations of ATR and 2 with CHK1 pathway mutations receiving corresponding inhibitors. The median number of cycles administered was 3 (range 1-72 cycles).

**Figure 2:**
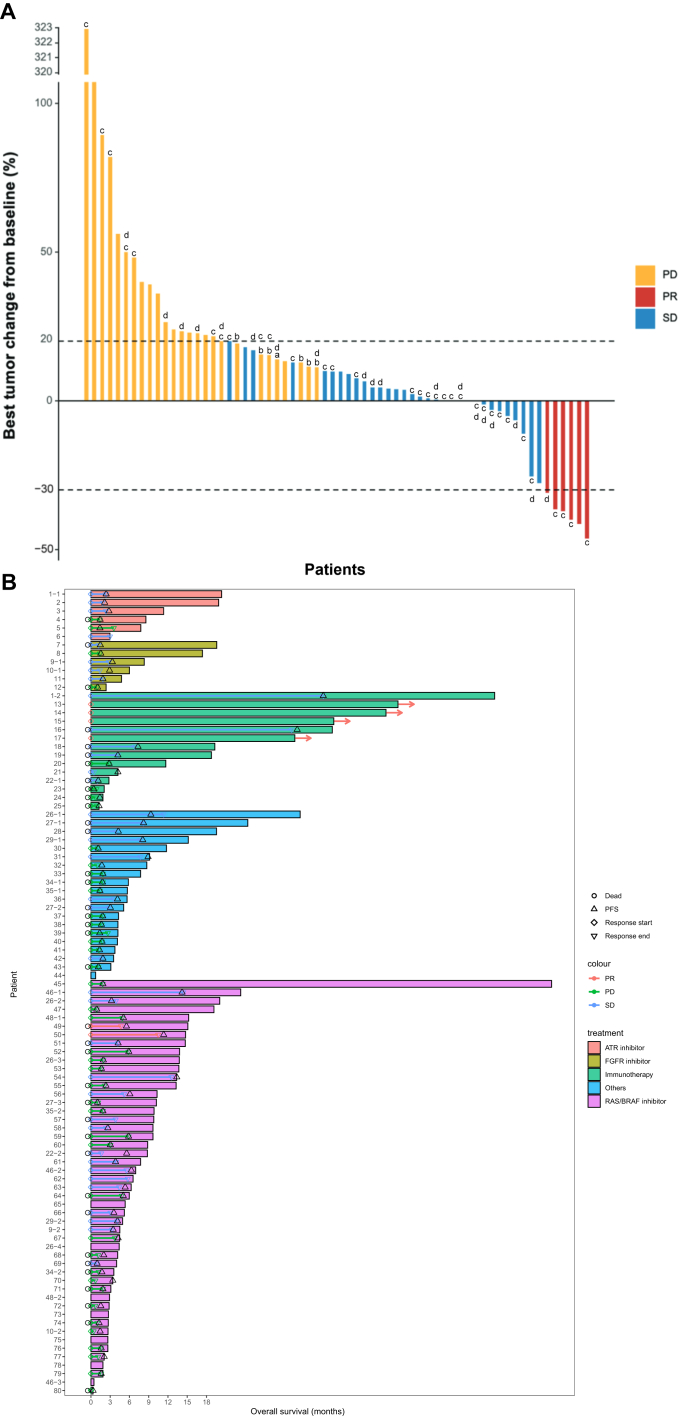
(A) Waterfall plot of each patient assessable for response according to RECIST 1.1. ^a^Progression due to occurrence of new lesions. ^b^Progression in non-target lesions. ^c^Patients who received recommended phase II dosing. ^d^Patient participating in more than one trial. (B) Integrated swimmer plot of overall survival with symbols for progression-free survival and duration of response, organized by treatment type, including those participating in more than one trial (denoted by *n*−1, *n*−2, etc.). ATR, ataxia-telangiectasia and Rad3-related protein; FGFR, fibroblast growth factor receptor; PD, progressive disease; PFS, progression-free survival; PR, partial response; RAS, rat sarcoma viral oncogene homolog; SD, stable disease.

Median follow-up across trials was 7.2 months (range 0.3-64.6 months). Up to 87 patients were assessable for response by RECIST v1.1. No complete responses were observed, and a partial response was seen in six (7%) patients making the ORR 7% across all trials. CBR was 51%. The waterfall plot designates the best overall response for each assessable patient (Figure 2).

Median OS of the total population was 16.8 months [95% confidence interval (CI) 8.8-22.0 months]. The 12-month survival rate was 58%. Median PFS was 2.6 months (95% CI 1.7-3.5 months). Kaplan–Meier survival curves for the overall population are shown in Supplementary Figure S1A and B, available at https://doi.org/10.1016/j.esmogo.2024.100054.

Univariable and multivariable regression was carried out to determine baseline demographic, clinical and biochemical values which were associated with longer OS. These were chosen on the basis of known prognostic factors based on existing literature and data availability (Supplementary Table S2, available at https://doi.org/10.1016/j.esmogo.2024.100054). Parameters with significant associations are shown in Table 2 for OS and Supplementary Table S3, available at https://doi.org/10.1016/j.esmogo.2024.100054, for PFS. Absence of adjuvant therapy and surgery for the primary tumor were associated with significantly shorter OS in the univariable analysis. Right-sidedness, presence of lung metastases and absence of surgery or adjuvant therapy also predicted poorer PFS. These characteristics remained negative predictors of prognosis, together with a low lymphocyte/monocyte ratio in the multivariable analysis (Table 2). A list of all variables analyzed is available in Supplementary Table S2, available at https://doi.org/10.1016/j.esmogo.2024.100054.

**Table 2:** Univariable and multivariable analyses of overall survival

Variable	*n*	Univariable analysis	Multivariable analysis		
		***P***	**HR**	***P***	**95% CI**
Age, years (<50 versus ≥50)	80	0.098	0.53	0.363	0.14-2.08
Surgery for primary tumor (yes versus no)	80	<0.001	0.05	0.025	0.004-0.69
Adjuvant therapy (yes versus no)	80	0.006	0.52	0.521	0.07-3.87
Tumor sidedness (right versus left)	56	0.840	0.10	0.0204	0.14-0.70
Liver metastases (no versus yes)	80	0.055	9.06	0.063	0.88-92.56
MSI status (low versus high)	35	0.063	3.13	0.378	0.25-39.41
Low lymphocyte/monocyte ratio (>2.8 versus ≤2.8)	80	0.5401	0.445	0.035	0.20-0.95

CI, confidence interval; HR, hazard ratio; MSI, microsatellite instability.

### Subgroup analysis

Survival outcomes were also assessed for patients receiving the recommended phase II doses (RP2D) and for the therapeutic group including those selected for trial by the presence of biomarkers.

Fifty-two patients received the actual RP2D, and 49 of them were assessable for responses. For this subgroup, ORR and CBF were 8% and 49%, respectively. Median OS for patients receiving the RP2D as opposed to those who did not was 14.7 months and 10.2 months, respectively (*P* = 0.87).

Survival outcomes for each therapeutic group and the ‘biomarker-selected’ population are given in Table 3. ORR for the biomarker-selected group was 15% and CBF 56%, compared with 7% and 51%, respectively, for the total population. Immunotherapies demonstrated a higher and longer benefit with a pooled median PFS of 5.8 months compared with 1.8, 2.1 and 4.2 months for FGFR inhibitors, ATR inhibitors and RAS/RAF inhibitors, respectively. The *P* value for difference between the populations was significant at *P* = 0.002. Kaplan–Meier survival curves for the subgroups are shown in Supplementary Figure S1C and D, available at https://doi.org/10.1016/j.esmogo.2024.100054. The swimmer plot in Figure 2B provides a graphical depiction of response and survival outcomes by therapeutic class for the entire patient population.

**Table 3:** Response and survival outcomes for treatment subgroups

Treatment	Subgroup	*n*	ORR (%)	CBR (%)	Median PFS (months, 95% CI)	Median OS (months, 95% CI)
RAS/RAF pathway inhibitors	Overall	49	6	43	3.1 (1.9-3.8)	13.2 (4.7-NE)
	BRAF mut	24	10	48	4.2 (2.1-66.3)	15.1 (12.8-17.4)
ATR inhibitor		6	0	67	2.1 (1.0-3.3)	NR
FGFR inhibitor		6	0	67	1.5 (1.1-2.0)	17.6 (2.1-NE)
Immunotherapies	Overall	14	29	71	4.2 (0.0-9.7)	33.8 (1.7-NE)
	MSI-H	7	43	86	1.8 (1.4-71.0)	NR
Other		20	0	42	1.8 (1.7-2.0)	10.5 (5.3-17.6)
Biomarker-selected group		37	15	56	2.37 (1.5-3.3)	16.9 (7.1-NE)
Overall population		95	7	51	2.6 (1.7-3.5)	16.8 (8.8-22.0)

ATR, ataxia-telangiectasia and Rad3-related protein; CI, confidence interval; FGFR, fibroblast growth factor receptor; HR, hazard ratio; MSI-H, microsatellite instability-high; NE, not estimable; NR, not reached; RAF, v-raf murine sarcoma viral oncogene homolog B1; RAS, rat sarcoma viral oncogene homolog.

## Discussion

In this study we demonstrated meaningful survival outcomes in patients with chemorefractory CRC with an OS of 16.8 months and a PFS of 2.6 months. These values are at least comparable with those reported for third-line agents and suggest that phase I trials might offer early access to effective therapeutics that could become a future standard of care.

A common requirement for entry on to phase I trials is a life expectancy ≥3 months. Best evidence in the late-line setting for CRC suggests a median OS of 5.0-5.3 months for placebo, and 6.4-7.1 months for final-line options of regorafenib and TAS-102.^14^^,^^15^ Despite the limitations of an indirect comparison, our trial population did markedly better, reaching a median OS of 13.6 months. This implies that phase I trials are a very reasonable choice for a fit population with refractory cancers, underscoring the importance of early patient referral. Furthermore, while selection bias might have affected the survival outcomes seen in our study due to the adoption of more stringent criteria to access treatment in a phase I setting, it may also be indicative of benefit to phase I participation that goes beyond assessments of ORR or CBR. These latter assessments showed low response rates in the overall population, as expected from a heavily pre-treated population as ours, with nearly a third having received more than two lines of treatment. Access to molecularly tailored treatments and administration of R2PD were evaluated as potential additional determinants of benefit. Indeed, while not compared explicitly, a biomarker-selected population did appear to respond better with an ORR of 15% and a CBR of 56%. This emphasizes the importance of understanding cancer biology in devising targeted therapeutics and the value of molecular characterization in early phase trials.^16^ In contrast, while also not compared directly, response rates in those who were administered the RP2D did not differ significantly from those in the overall population. The discrepancy between response rates and OS suggests the need for improved methods of response measurement, especially as it is increasingly recognized that high response rates may not be reasonable in a heavily pre-treated population, and may not capture the long-term benefit from immunotherapy and targeted treatments.^17^, ^18^, ^19^

**Figure 3:**
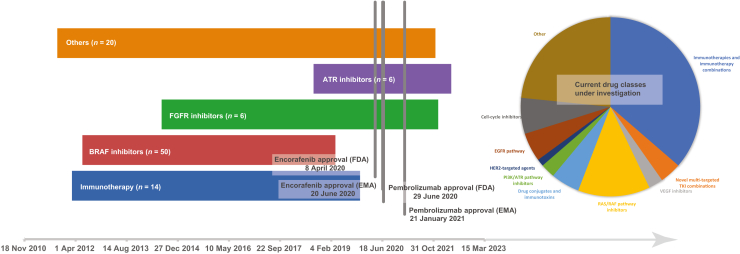
**Timeline of patient recruitment to molecularly targeted therapies against regulatory approval of these therapies in the UK, Europe and the United States.** Pie chart represents agent classes currently under active investigation according to clinicaltrials.gov data (see Supplementary Table S2, available at https://doi.org/10.1016/j.esmogo.2024.100054, for details). ATR, ataxia-telangiectasia and Rad3-related protein; EGFR, epidermal growth factor receptor; EMA, European Medicines Agency; FDA, Food and Drug Administration; FGFR, fibroblast growth factor receptor; RAF, v-raf murine sarcoma viral oncogene homolog B1; RAS, rat sarcoma viral oncogene homolog; VEGF, vascular endothelial growth factor.

A number of effective therapeutics have arisen for CRC treatment in recent years, each proceeding through phase I trials before later assessments of efficacy. Indeed, certain therapeutics have even obtained accelerated regulatory approval off the back of early phase testing,^20^ despite this usually requiring phase III assessment against standard of care. Access for our patients to these now standard options far preceded the corresponding regulatory approvals (Figure 3). Given that the phase I field for CRC is expanding rapidly, patients currently enrolled in early phase trials face the possibility of receiving treatments which may be approved within the next decade. Current targets of interest in CRC include combinations with immunotherapy, multi-targeted tyrosine kinase inhibitors, second- and third-generation BRAF inhibitors and other inhibitors of the RAS pathway, human epidermal growth factor receptor 2-targeted agents, as well as other molecular targets such as CDK and PI3K (Supplementary Table S4, available at https://doi.org/10.1016/j.esmogo.2024.100054).

Of note, MSI testing was not carried out on 56 (70%) of our patients, indicating the change in regulatory recommendations for testing that followed tumor agnostic approval of pembrolizumab in 2017.^6^^,^^20^ With a recruitment period that began in 2011, many of our patients would not have had access to routine testing for microsatellite stability. However, given that pooled analyses consistently suggest issues with uptake of molecular diagnostics across multiple tumor types,^21^ this is unlikely to affect the generalizability of our results. While ideally frontline testing of tumors for molecular alterations should be available, in resource-restricted settings, phase I studies with built-in diagnostics beyond a single predictive biomarker may provide benefit beyond recruitment to the trial in question.

Our univariable and multivariable analyses are in agreement with current literature around a shorter OS for right-sided tumors.^22^ In spite of the good performance status and lack of comorbidities seen with younger patients,^23^, ^24^, ^25^ there was no detectable difference between younger and older patients for either PFS or OS, acknowledging that older patients were highly selected. An improved survival for those undergoing surgery on the primary tumor could be attributable to differences between synchronous metastatic disease and recurrent disease, the latter possibly suggesting greater indolence, as well as avoidance of morbidity arising from malignant bowel obstruction or perforation. However, despite synchronous metastatic disease being predictive of worse survival in other tumor types,^26^ this has not been clearly proven in CRC,^27^ and data are conflicting about the role of palliative surgery on the primary tumor in the setting of metastatic disease.^28^^,^^29^

Our study acknowledges a number of limitations. Our population was a heterogeneous cohort of patients receiving a different range of novel therapeutics in a late-line setting and therefore interpretation of the response assessment is difficult, as is the attribution of treatment effect in the absence of a control group. Additional heterogeneity was introduced by the evaluation of patients enrolled in different dose-level cohorts, as expected in dose-escalation trials, and having received variable prior lines of therapy. Moreover, our means of dealing with patients participating in multiple trials, while necessary, was not ideal for survival analysis. For context, subsequent outcomes of patients participating in multiple trials is given in Supplementary Table S5, available at https://doi.org/10.1016/j.esmogo.2024.100054. Despite these limitations, this study provides a more contemporary assessment of outcomes in early phase trials,^30^^,^^31^ especially within a population where demand for trial options is increasing commensurate with a rising incidence of malignant diagnoses.

Data from clinical trials, even those that are early and potentially fail to meet efficacy endpoints, comprise large repositories of high-quality clinical and tumor information from which pooled and retrospective assessments such as those we present here can generate new knowledge about prognosis and treatment response beyond the question that they were designed to answer. Further research should be directed at making use of these repositories to inform clinical trial design and biomarker selection.

## Conclusion

Phase I trials provide substantial benefits for patients with refractory CRC in allowing access to potentially efficacious treatment options and molecular testing that may not otherwise be available. Priority should be given to early consideration of phase I involvement before exhausting late-line conventional treatments. Using high-quality patient information may contribute to inform future trial design and patient selection.
