# Supplementary material for: Benefits from early trial involvement in metastatic colorectal cancer: outcomes from the phase I unit at the Sarah Cannon Research Institute UK
Source: ESMO Gastrointest Oncol. 2024 Apr 17;4:100054. doi: 10.1016/j.esmogo.2024.100054 (PMC12836528; doi:10.1016/j.esmogo.2024.100054)
Supplement: Supplementary Table S1 [file mmc1.docx]

| **Molecular NGS testing for CRC-related oncogenes Molecular Profile (n=30)** | **n (%)** |
| --- | --- |
| *ERBB2* mutation (n=27) | |
| Yes | 1 (3.7) |
| No | 26 (96.3) |
| *PIK3CA* mutation (n=30) | |
| Yes | 11 (36.7) |
| No | 19 (63.3) |
| *ATM* mutation (n=20) | |
| Yes | 3 (15) |
| No | 17 (85) |
| *MET* mutation * (n=27) | |
| Yes | 1 (3.7) |
| No | 26 (96.3) |
| *AKT1* mutation (n=27) | |
| Yes | 1 (3.7) |
| No | 26 (96.3) |
| *BRCA1* mutation (n=19) | |
| Yes | 1 (5.3) |
| No | 18 (94.7) |
| *BRCA2* mutation (n=19) | |
| Yes | 1 (5.3) |
| No | 18 (94.7) |
| *FGFR* alteration (n=27) | |
| Yes | 2 (7.4) |
| No | 25 (92.6) |
| *NTRK* fusions (n=3) | |
| Yes | 0 (0) |
| No | 3 (100) |
| *TP53* mutation (n=30) | |
| Yes | 24 (80) |
| No | 6 (20) |
| *PTEN* mutation** (n=27) | |
| Yes | 3 (11.1) |
| No | 24 (88.9) |
| TMB score (n=19) | |
| TMB High (>10) | 5 (26.3) |
| TMB Low (≤10) | 12 (63.2) |
| NA | 2 (10.5) |
