# Supplementary material for: Benefits from early trial involvement in metastatic colorectal cancer: outcomes from the phase I unit at the Sarah Cannon Research Institute UK
Source: ESMO Gastrointest Oncol. 2024 Apr 17;4:100054. doi: 10.1016/j.esmogo.2024.100054 (PMC12836528; doi:10.1016/j.esmogo.2024.100054)
Supplement: Supplementary Table S2 [file mmc2.docx]

| **Patient characteristics** | **Treatment characteristics** | **Tumor characteristics** | **Biochemical characteristics** |
| --- | --- | --- | --- |
| Age  Sex  ECOG | Surgery for primary tumor  Surgery for metastasis  Adjuvant therapy  Neo-adjuvant therapy  Number of prior lines of therapy  Duration of oxaliplatin  Duration of irinotecan  Treatment received  RP2D | Stage at diagnosis  Histological type  Tumor grade  Tumor sidedness  Tumor location  RAS mutation  BRAF mutation  RAS/RAF wildtype  Liver metastases  Lung metastases  Bone metastases  Lymph node metastases  Brain metastases  Peritoneal metastases  Number of metastatic sites  MSI status | Platelet count  Haemoglobin  LDH  ALP  ALT  AST  Lymphocyte-to-monocyte ratio  Neutrophil-to-lymphocyte ratio  WBC  Albumin |
