# Supplementary material for: Benefits from early trial involvement in metastatic colorectal cancer: outcomes from the phase I unit at the Sarah Cannon Research Institute UK
Source: ESMO Gastrointest Oncol. 2024 Apr 17;4:100054. doi: 10.1016/j.esmogo.2024.100054 (PMC12836528; doi:10.1016/j.esmogo.2024.100054)
Supplement: Supplementary Table S3 [file mmc3.docx]

| **Molecular therapy** | **Active trials** |
| --- | --- |
| Immunotherapies and immunotherapy combinations | NCT04929223  NCT03626922  NCT03374254  NCT04991948  NCT05019534  NCT03657641  NCT04550897  NCT04468607  NCT05627635  NCT04599140  NCT04017650  NCT03436563  NCT03712943  NCT04362839  NCT05609370  NCT03798626  NCT03290937  NCT03377361  NCT04294160  NCT04140526  NCT03866239  NCT04014530  NCT04606472  NCT05162755  NCT04208958  NCT03761017  NCT04666688  NCT05620134 |
| Novel multi-targeted TKI combinations | NCT04873895  NCT03724851  NCT04868773 |
| VEGF inhibitors | NCT04073615  NCT05155124 |
| RAS/RAF pathway inhibitors | NCT02613650  NCT05497336  NCT02188264  NCT05039177  NCT03317119  NCT04616183  NCT05200442  NCT04449874  NCT04916236  NCT05585320 |
| Drug conjugates and immunotoxins | NCT03983954  NCT04410224  NCT05001282  NCT04171141 |
| PI3K/ATR pathway inhibitors | NCT04495621  NCT03711058 |
| HER2 targeted agents | NCT05382364 |
| EGFR pathway | NCT05379595  NCT03526835  NCT04844073  NCT05783622 |
| Cell-cycle inhibitors | NCT05093907  NCT03714958  NCT05375604  NCT03263429  NCT02649790 |
| Other* | NCT05743036  NCT05167448  NCT04660812  NCT05379595  NCT04826003  NCT05205330  NCT05497336  NCT04328740  NCT03597581  NCT05107674  NCT05360680  NCT05759923  NCT04535401  NCT05397171  NCT04408599  NCT04916236  NCT02632448  NCT03337087 |

*Includes WEE1 inhibitors, DLL4, adenosine receptor inhibitors, MET inhibitors, FAP-inhibitors, EP4 receptor inhibitors, pyruvate kinase inhibitor, creatine kinase-B inhibitors, CBL-B inhibitors, WT1 inhibitors, arginase inhibitors, ATR inhibitors, GDF-15 inhibitors, anti-LAIR-1, SHP-2 inhibitors, CHK-1 inhibitors, and PARP inhibitors.

**Table S3** Current targets in phase I testing according to registration with the clinicaltrials.gov database. Search strategy involved use of keywords “colorectal cancer” AND (“phase I” OR “phase I/II”) AND “active” AND (“adult” OR “older adult”). Trials were eligible for inclusion if they assessed a molecularly targeted agent or immunotherapy either as monotherapy or in combination with chemotherapy. Trials were excluded if they assessed vaccines, or non-targeted technologies such as radiotherapy, TACE or IPEC.
