# Supplementary material for: Benefits from early trial involvement in metastatic colorectal cancer: outcomes from the phase I unit at the Sarah Cannon Research Institute UK
Source: ESMO Gastrointest Oncol. 2024 Apr 17;4:100054. doi: 10.1016/j.esmogo.2024.100054 (PMC12836528; doi:10.1016/j.esmogo.2024.100054)
Supplement: Supplementary Table S5 [file mmc4.docx]

| Patient | Treatment | PFS (months) | Reference |
| --- | --- | --- | --- |
| 1 | Other | 0.93 | 1.68 (1.47-3.84) |
| 1 | Other | 2.73 | 1.68 (1.47-3.84) |
| 2 | RAS/RAF | 2.61 | 3.06 (1.8-4.53) |
| 3 | RAS/RAF | 1.53 | 3.06 (1.8-4.53) |
| 4 | RAS/RAF | 5.67 | 3.06 (1.8-4.53) |
| 4 | RAS/RAF | 0.42 | 3.06 (1.8-4.53) |
| 5 | RAS/RAF | 2.88 | 3.06 (1.8-4.53) |
| 5 | RAS/RAF | 1.74 | 3.06 (1.8-4.53) |
| 5 | RAS/RAF | 3.96 | 3.06 (1.8-4.53) |
| 6 | RAS/RAF | 3.72 | 3.06 (1.8-4.53) |
| 7 | ATR | 2.13 | 1.92 (1.26-NE) |
| 8 | RAS/RAF | 1.29 | 3.06 (1.8-4.53) |
| 9 | RAS/RAF | 3.12 | 3.06 (1.8-4.53) |
| 10 | Other | 1.26 | 1.68 (1.47-3.84) |
| 11 | IO | 1.02 | 28.92 (1.14-NE) |

**Table S5** Details of therapies received and progression free survival of patients receiving further lines of subsequent phase I therapy. Reference is the remaining population following exclusion of these patients according to treatment class.
