# Supplementary material for: Benefits from early trial involvement in metastatic colorectal cancer: outcomes from the phase I unit at the Sarah Cannon Research Institute UK
Source: ESMO Gastrointest Oncol. 2024 Apr 17;4:100054. doi: 10.1016/j.esmogo.2024.100054 (PMC12836528; doi:10.1016/j.esmogo.2024.100054)
Supplement: Supplementary Table S4 [file mmc6.docx]

|  |  | **Univariable analysis** | **Multivariable analysis** | | |
| --- | --- | --- | --- | --- | --- |
| **Variable** | **N** | **p** | **HR** | **p** | **95% CI** |
| Surgery for primary tumor (yes vs no) | 96 | 0.017 | 0.70 | 0.37 | 0.32-1.53 |
| Adjuvant therapy (yes vs no) | 96 | 0.043 | 0.67 | 0.22 | 0.35-1.28 |
| Tumor sidedness (right vs left) | 67 | 0.013 | 2.01 | 0.02 | 1.12-3.61 |
| Lung metastasis (yes vs no) | 96 | 0.031 | 1.59 | 0.11 | 0.90-2.80 |

Supplementary table 4: Univariate and multivariate analysis of progression-free survival.
